# Supplementary material for: Passive detection of COVID-19 with wearable sensors and explainable machine learning algorithms
Source: NPJ Digit Med. 2021 Dec 8;4:166. doi: 10.1038/s41746-021-00533-1 (PMC8655005; doi:10.1038/s41746-021-00533-1)
Supplement: Supplementary file 1 — Supplementary Information [file 41746_2021_533_MOESM1_ESM.pdf]

Passive Detection of COVID-19 with Wearable Sensors and Explainable Machine Learning Algorithms

Matteo Gadaleta, Jennifer M. Radin, Katie Baca-Motes, Edward Ramos, Vik Kheterpal, Eric J. Topol, Steven R. Steinhubl, Giorgio Quer

SUPPLEMENTARY INFORMATION

True positives (TP), true negatives (TN), false positives (FP) and false negatives (FN) for the models at the optimal operating points are reported in Supplementary Table 1.

Analysis in the absence of self-reported symptoms

For an improved and effective tracking of the pandemic, many research institutions are collecting sensor data from individuals, while it is not always possible to provide surveys and collect active feedback from participants. Information actively added by participants may be crucial especially for potentially infected individuals, indeed a fully passive data collection system may be adopted by a broader audience and have a more capillary diffusion.

Our model can be leveraged to support also these studies based uniquely on passive data collection. To this end, we performed an additional analysis without the inclusion of self-reported symptoms as source of knowledge for the model. The results of the analysis without considering self-reported symptoms are shown in terms of AUC of the ROC. (Supplementary Figure 1)

	Excluding data after COVID-19 Test		Including data after COVID-19 Test	
	Symptomatic	No Symptom Reported	Symptomatic	No Symptom Reported
TP	338 [317 - 360]	328 [305 - 350]	403 [382 - 422]	372 [348 - 394]
TN	960 [925 - 996]	5533 [5454 - 5611]	1018 [983 - 1052]	5885 [5810 - 5959]
FP	560 [524 - 595]	2310 [2232 - 2389]	502 [468 - 537]	1958 [1884 - 2033]
FN	201 [179 - 222]	264 [242 - 287]	136 [117 - 157]	220 [198 - 244]

Supplementary Table 1 – True positives (TP), true negatives (TN), false positives (FP) and false negatives (FN) for the models at the optimal operating points. Results for symptomatic and no-symptom-reported cohorts, both excluding and including data after the COVID-19 test are reported. 95% confidence intervals are reported in brackets.

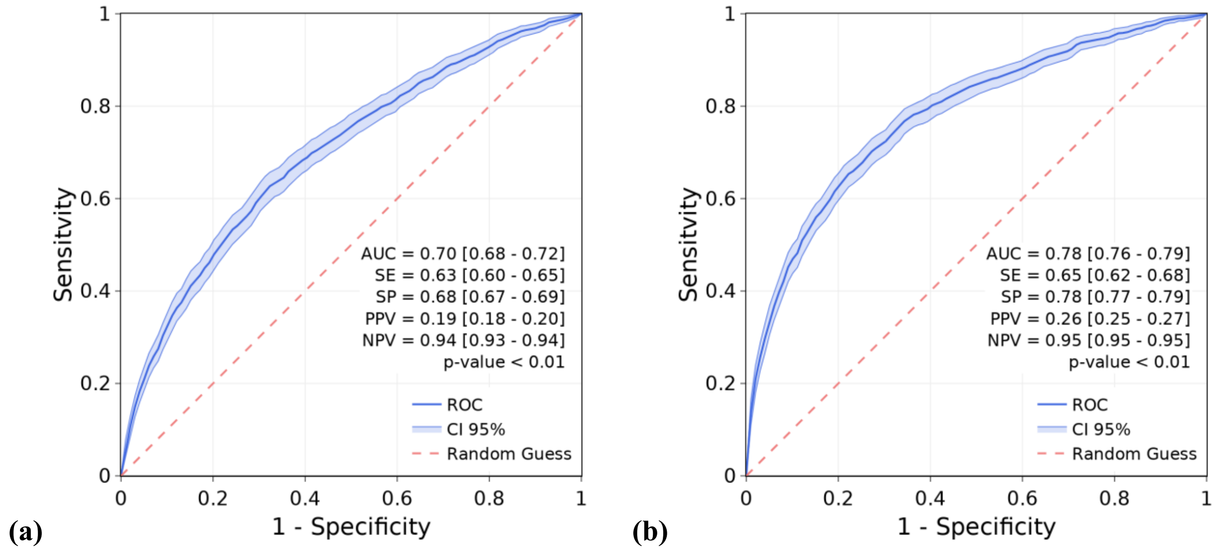

Supplementary Figure 1 – Receiver operating characteristic curves (ROCs) for the discrimination between COVID-19 positive and COVID-19 negative when the self-reported symptom data is not used in the prediction. Performance obtained by excluding (a) and including data after the test date (b) are reported. The model is a gradient boosting prediction model based on decision trees. Median values and 95% confidence intervals (CIs) for sensitivity (SE), specificity (SP), positive predictive value (PPV) and negative predictive value (NPV) are reported, considering the point on the ROC with the highest average value of sensitivity and specificity. Error bars represent 95% CIs. p-values of the one-sided Mann-Whitney U test are reported.
